# Rewilding: a requirement for a sustainable future

**DOI:** 10.1186/s40850-023-00187-4

**Published:** 2023-11-03

**Authors:** Ashish Kumar Arya

**Affiliations:** Department of Environmental Science, Graphic Era (Deemed to be University), Dehradun, India

## Abstract

Nowadays rewilding has received an increasing focus as a sustainable conservation tool for restoring damaged or disturbed habitats. Many types of rewilding initiatives have been implemented all over the globe with the goal of reinstalling the extinct or lost fauna as well as restoring ecological relationships and natural processes. The effectiveness of rewilding initiatives depends on a comprehensive understanding of the ecological functions, habitat needs, social behaviour, and interrelation among various animal species. This Collection invites contributions that portray different aspects and the importance of rewilding.

## Main text

The entire globe is facing the very serious challenge of biodiversity loss with a significant number of species facing extinction due to various anthropogenic disturbances including habitat loss, overexploitation, pollution, climate change and invasive species [2, 4]. In June 2021 the United Nations (UN), therefore, declared the decade of ecosystem restoration.

Ecological restoration has become an effective tool for reversing environmental degradation, reducing climate change, and recovering lost biodiversity as well as crucial ecosystem services. Approaches for conserving and restoring natural habitats supported by ecologists and conservationists in the past, included rewilding, restoration, and reintroduction.

The process of rewilding is an approach that aims to rehabilitate ecosystems and prevent biodiversity loss by allowing wild animals and natural processes to reclaim places no longer managed by human beings [1]. The word rewilding was initially introduced for the restoration of interconnected natural regions that greatly supported wild animal species in the north American region approximately three decades ago [2]. Rewilding of any area directly and indirectly depends on three main components namely (i) large, protected core reserves (ii) connectivity (iii) and keystone species [3].

The rewilding approach restores and recovers the natural resources and connectivity of degraded ecosystems, through the reintroduction of extinct species or the reduction of human interference [4]. Re-wilding also aims to increase biodiversity and complexity of an ecosystem by allowing nature to take its course.

The term restoration was coined by Aldo Leopold in 1949 and is defined as the art of making things natural again. In other words, restoration is an effective tool to re-establish the habitat that has been degraded and damaged by different types of interference. This approach usually restores the function, composition and structure of an ecosystem [5]. The main aim of restoration is to enhance the ecosystem productivity by applying natural practices.

The term reintroduction was first described by George Rabb in 1976 and is defined as an approach to relocate species from another area to their native range, where they have become rare or extinct [6]. The main aim of the reintroduction is to restore the species abundance, diversity and distribution in its native ranges.

All three above mentioned concepts are interconnected but their aims, methodologies and outputs are different. However, they can sustain and strengthen each other in various ways. For instance, rewilding can support the restoration practice to increase the adaptability, complexity and diversity of an ecosystem [7]. Restoration can promote rewilding by providing suitable habitats and pathways for species reintroduction [8]. In a similar way, restoration can contribute to rewilding by restoring key ecological links, such as predation or herbivory [9]. Furthermore, rewilding may support restoration by providing the suitable environmental conditions, such as reduced human interference and enhanced food availability [10].

However, these interventions create some challenges that must be carefully considered and managed. For example, rewilding can lead to conflicts with human interests and activities, such as agriculture, forestry, hunting, fishing and development [4]. In addition, restoration can negatively impact local livelihoods and well-being, such as loss of income, resources, access, security and identity. For example, the removal of an ecologically harmful industrial project to restore a particular landscape directly affects local livelihoods and well-being [8]. In a similar way, reintroductions can raise ethical and legal issues about the rights and responsibilities of humans and animals in redeveloped areas [4]. Furthermore, rewilding can face ecological uncertainties and challenges, such as climate change, invasive species, diseases and human intervention [10].

These interventions consequently require a holistic approach and balance adaptations to natural and human needs and values. It is about engaging with different stakeholders to understand their perspectives and aspirations, identifying shared goals and objectives that are consistent with conservation priorities, creating a participatory process to ensure transparency and accountability, implement flexible business practices that monitor and evaluate effects and outcomes, and communicate effectively to provide foster on insight and support [2, 9].

These approaches have the potential of achieving triple conservation success: biodiversity enhancement, ecosystem restoration, and species reintroduction.

In this Collection, we will explore the important topics of Rewilding by inviting authors for contribution from across the globe. This will provide a comprehensive and balanced research perspective and enable potential ideas about restoration and reintroduction.

## Data Availability

Not Applicable.

## Abbreviations

UN: United Nation
